# Subtrochanteric periprosthetic femoral fracture with a resurfacing metal-on-metal prosthesis in situ in an elderly patient: An increasing problem? – A case report and literature review

**DOI:** 10.1016/j.tcr.2025.101285

**Published:** 2025-11-17

**Authors:** Luca H. Plekkenpol, Judith Olde Heuvel, A. Sybrand Homan, Roy B.G. Brokelman, Bas L. Fransen

**Affiliations:** a Department of Orthopaedics, OCON Orthopedische en Sportmedische kliniek, Geerdinksweg 141, 7555 DL, Hengelo, the Netherlands; b Faculty of Medical Sciences, University of Groningen, Antonius Deusinglaan 1, 9713 AV, Groningen, the Netherlands

**Keywords:** Hip resurfacing, Subtrochanteric fractures, Periprosthetic fractures, Geriatric patients

## Abstract

**Aims:**

Subtrochanteric periprosthetic femoral fractures (PPFF) in elderly patients with metal-on-metal hip resurfacing implants are rare and pose significant management challenges. This case report describes the surgical management of such a fracture and provides a review of reported treatment strategies.

**Methods:**

A 79-year-old woman with a sixteen-year-old Birmingham Hip Resurfacing (BHR) prosthesis sustained a comminuted subtrochanteric femoral fracture following a fall. The fracture was treated with open reduction and internal fixation (ORIF) using a Locking Compression Plate and cerclage wires. A literature review was conducted to evaluate surgical approaches and outcomes for similar cases.

**Results:**

Postoperatively, the patient mobilized with weight-bearing restrictions and showed satisfactory fracture healing at follow-up, with radiographic consolidation at four months and full functional recovery. The literature review showed that ORIF, primarily using plate osteosynthesis, is the preferred treatment for subtrochanteric PPFFs in resurfacing arthroplasty, yielding good fracture consolidation and mobility outcomes. Total hip arthroplasty (THA) is typically reserved for fractures with implant loosening or insufficient bone quality.

**Conclusion:**

ORIF is an effective treatment for subtrochanteric PPFF in elderly patients with stable resurfacing implants, promoting fracture healing and functional restoration. This case underscores the complexity of managing such fractures in aging populations and emphasizes the need for individualized treatment plans. Given the aging population with resurfacing prostheses, further research is needed to optimize treatment strategies and improve patient outcomes.

## Highlights

• Rising incidence of periprosthetic femoral fractures (PPFF) in geriatric patients.
• ORIF effective for subtrochanteric PPFF in elderly with stable resurfacing implant.
• Increased morbidity in elderly patients require tailored treatment strategies.
• Total Hip Arthroplasty used for PPFF with implant loosening or poor bone quality.

## Introduction

Metal-on-metal (MoM) resurfacing hip prostheses, including the Birmingham Hip Resurfacing (BHR) implant, were developed in the 1990s, to address the early failure and subsequent high revision rates of previous MoM total hip replacement (THR) implants. Despite controversy [1], satisfactory clinical outcomes regarding mid- to long-term survival and improved function have been demonstrated [1,2]. The benefits of a bone sparing procedure, improved biomechanical restoration and increased stability have contributed to an increased use of BHR implants in younger patients (under 55 years old) with osteoarthritis of the hip and higher physical demands [1,3]. Consequently, there has been a corresponding rise in periprosthetic fractures located in the femoral neck and the trochanteric region as these patients age [3,4]. Incidences in the first year after MoM resurfacing have been reported to be around 1–2% [3], but long-term fracture rates in this specific group are not well known.

Traumatic fractures, specifically subtrochanteric periprosthetic femoral fractures (PPFF) in the presence of a BHR arthroplasty, will require optimized treatment approaches in elderly patients, but have rarely been described in literature [3,5]. Moreover, the existing literature on this type of fracture mainly concerns patients aged between thirty to fifty years [2,6,7]. The aim of this report was to describe the treatment of a subtrochanteric PPFF in a 79-year-old female patient with a BHR prosthesis in situ, and discuss the literature published in relation to this subject.

## Case description

**Fig. 1:**
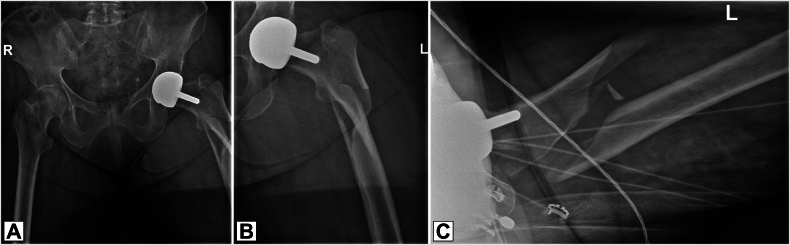
Preoperative radiographs, showing a left subtrochanteric femoral fracture with slight dislocation, distal to a Birmingham Hip Resurfacing prosthesis. A: Anterior-Posterior (AP) X-ray of pelvis. B: AP X-ray of left femur. C: Axial X-ray of left femur.

A 79-year-old woman presented to the emergency department with left hip pain following a fall. She had a history of hip osteoarthritis and underwent BHR MoM resurfacing sixteen years ago. X-rays revealed a comminuted subtrochanteric femoral fracture (AO 31A3.3) (Fig. 1). The patient's pre-existing physical condition was good; she ambulated without the use of walking aids and lived independently. The patient had a history of ischemic stroke, was on anticoagulants (apixaban), and had reduced kidney function and a previous total knee arthroplasty. During admission, she required blood transfusions, which led to an adverse reaction, delaying surgery by six days.

Surgery involved a posterolateral approach for open reduction and internal fixation (ORIF), using an inverted distal femur 4.5 mm Locking Compression Plate (LCP; DePuy Synthes, West Chester, Pennsylvania, USA), carefully fixated with two fully threaded cortical screws and a partially threaded cancellous screw, ensuring no impingement on the prosthesis stem. The plate was locked distally with multiple cortical screws and cerclage wires were used to supplement the construct. Fracture reduction and screw positioning were confirmed with fluoroscopy.

**Fig. 2:**
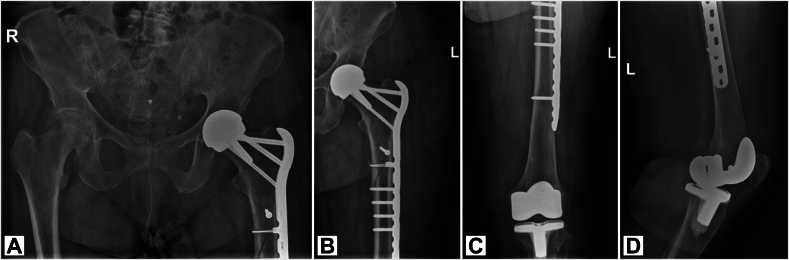
Postoperative radiographs at six weeks after surgery, showing the prosthesis and the Locking Compression Plate with cortical screws and cerclages. The femur is in anatomical position and shows signs of callus formation at the height of the fracture. A: Anterior-Posterior X-ray of Pelvis. B: Lauenstein X-ray of proximal femur. C: AP X-ray of distal femur. D: Lateral X-ray of left knee, showing a total knee arthroplasty.

**Fig. 3:**
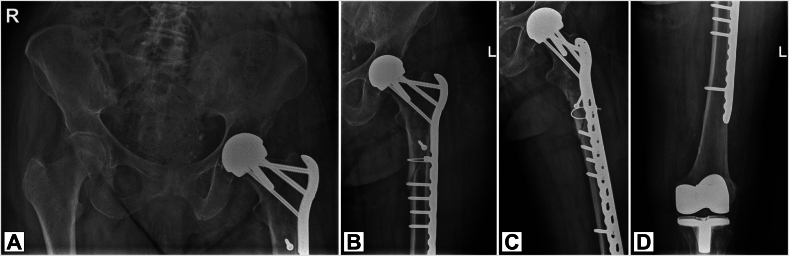
Postoperative radiographs, four months after reduction and fixation of subtrochanteric femoral fracture with a Locking Compression Plate, cerclage wires and multiple cortical screws, showing signs of callus formation. A: Anterior-Posterior (AP) X-ray of pelvis. B: AP X-ray of proximal left femur. C: Lauenstein X-ray of left femur. D: AP X-ray of distal left femur.

Postoperatively, the patient was able to mobilize unloaded with a walking aid and was discharged to a geriatric rehabilitation care facility, adhering to non-weight bearing restrictions for six weeks due to the osteoporotic nature of her bone observed during surgery. X-rays assessment at six weeks (Fig. 2) postoperatively was satisfactory, allowing her to progress to fifty percent weight bearing. X-rays at four months showed fracture consolidation (Fig. 3). She had no residual pain and she recovered functionally satisfactorily, regaining her pre-injury capabilities. This was confirmed again during final follow-up at six months.

## Discussion

This case report details the successful treatment of a subtrochanteric periprosthetic femoral fracture in a 79-year-old woman with a well-functioning Birmingham Hip Resurfacing implant in situ for sixteen years. With the historical rise in hip resurfacing among younger patients in the early 2000s, an increase in such fractures is anticipated as this cohort ages. This report examines the challenges of treating traumatic periprosthetic fractures in elderly patients with long-standing BHR implants.

Subtrochanteric periprosthetic fractures with resurfacing prostheses are infrequently reported, with treatment varying by patient age and clinical context. In elderly patients, ORIF with plate osteosynthesis is prevalent. Lugani et al. (2021) reported about the treatment of a subtrochanteric periprosthetic fracture (type IV.3-C) in a 69-year-old male with a BHR implant in situ for twelve years [3]. In this case, synthesis was performed using a 4.5/5.0 LCP with trochanteric fixation. However, the rehabilitation was complicated by breakage of a proximal screw on the plate and varus collapse of the fracture, which was treated conservatively. Furthermore, Fraile Gamarra et al. (2019) described the case of an 81-year-old female patient with a subtrochanteric spiral fracture in the presence of a Birmingham Mid-Head Resection (BMHR) prosthesis [5]. The treatment entailed ORIF with a trochanteric plate and three cerclages and unloaded mobilizing with crutches for two months, leading to a satisfactory reduction of the fracture. Alternatively, Aning et al. (2005) treated a fracture in a 60-year-old male using an intramedullary nail, locking screws and cerclage wires. After six weeks of unloaded mobilizing with crutches, the patient achieved full recovery [6].

For younger patients, there were also different treatment modalities used. In the article of Peskun et al. (2012), an isolated subtrochanteric PPFF in a 41-year-old male was anatomically reduced in a closed manner on a traction table, followed by surgical management using a trochanteric start point cephalo-medullary nail [7]. Another case, published by Whittingham-Jones et al. (2010), concerns a 32-year-old female who endured a comminuted subtrochanteric femoral shaft fracture, accompanied by a contralateral superior pubic ramus fracture [2]. The femoral fracture was treated surgically using a broad Dynamic Compression Plate (DCP) and multiple cortical screws.

These cases highlight the variability in treatment approaches, but do not provide any resolution to the debate about the ideal osteosynthesis method. In most cases, plate osteosynthesis with ORIF was used, as well as in our case. This decision was also made in our case with the goal to aim for absolute stability of the fracture. Due to the biomechanical characteristics of the resurfacing prosthesis like sufficient residual bone stock, most cases with subtrochanteric periprosthetic femoral fractures are treated using osteosynthesis. Naturally, residual stability of the existing prosthesis is an important factor in the choice of treatment method [3].

Given patient-specific factors in the elderly population, such as an increased tendency to fall, an increased number of comorbidities [8,9], higher incidence of osteoporosis and frailty [5,9], the incidence of periprosthetic hip fractures is expected to rise by an average 4.6% per decade over the next 30 years [4]. Periprosthetic fractures in elderly patients with BHR implants pose challenges, due to osteoporosis and comorbidities, leading to greater vulnerability and thereby complicating both the fixation of fractures and the integration of implants. Achieving the mechanical stability required for healing may be difficult, increasing the risk of delayed or non-union. Furthermore, implanting a prosthesis into osteoporotic bone is associated with early subcapital fractures, besides surgical notching of the femoral neck, malposition of the prosthesis and the evolution of postsurgical avascular necrosis [2]. Additionally, higher age has been shown to be a risk factor for mortality and complications after orthopaedic hip surgery [8]. Post-operative complications such as delirium, lower respiratory tract infections and gastrointestinal bleeding occur more frequently. Moreover, deep vein thrombosis, urinary retention and aspiration pneumonia have been reported [9].

The BHR prosthesis itself also presents unique challenges. Preservation of bone stock is one of its advantages, but this also entails that any revision surgery must navigate around the existing prosthesis without compromising the remaining bone integrity. Neck narrowing, observed in patients with long-term BHR implants, may influence the decision between fracture fixation and revision [1]. Treating PPFF with a resurfacing prosthesis in situ demands careful planning, with ORIF and Total Hip Arthroplasty (THA) as primary options. ORIF is less invasive, preserves existing bone stock, and is associated with lower immediate postoperative morbidity, making it a viable option if anatomical reconstruction can be achieved. However, ORIF has a higher risk of reoperation and complications if the reconstruction is not perfect. In contrast, complete revision to THA provides a comprehensive solution, particularly effective for fractures involving loose stems. Nevertheless, THA is more invasive and associated with higher morbidity and immediate postoperative mortality [10].

Most patients are treated by ORIF, with good results in terms of fracture consolidation and mobility. THA could be a viable alternative in the case of loosening of the stem of the resurfacing prosthesis or insufficient bone quality.

This case report illustrates the successful treatment of a subtrochanteric periprosthetic fracture in a 79-year-old woman with a well-functioning BHR implant in situ, despite challenges posed by her age and comorbidities. Open reduction and internal fixation with a Locking Compression Plate successfully achieved fracture union and full functional recovery within a six-month follow-up period. However, this period, while sufficient to confirm healing and functional recovery, does not assess long-term prosthesis outcomes, and the limited sample size of similar cases restricts generalizability. This case emphasizes the importance of tailored treatment approaches and the necessity for further research with larger study populations to optimize management strategies for elderly patients with periprosthetic femoral fractures in the presence of resurfacing implants.

## CRediT authorship contribution statement

**Luca H. Plekkenpol:** Conceptualization, Formal analysis, Investigation, Project administration, Writing – original draft, Writing – review & editing. **Judith Olde Heuvel:** Methodology, Resources, Supervision, Writing – review & editing. **A. Sybrand Homan:** Data curation, Resources, Supervision, Writing – review & editing. **Roy B.G. Brokelman:** Conceptualization, Formal analysis, Investigation, Writing – review & editing. **Bas L. Fransen:** Conceptualization, Data curation, Investigation, Methodology, Supervision, Writing – review & editing.

## Statement of informed consent

Written informed consent was obtained from the patient for data collection, storage and publication, in accordance with institutional and ethical guidelines.

## Declaration of competing interest

The authors declare that they have no known competing financial interests or personal relationships that could have appeared to influence the work reported in this paper.

## Data Availability

Not applicable.
